# Psychotherapeutic approaches: hopefully, globally effective

**DOI:** 10.3389/fpsyt.2024.1322184

**Published:** 2024-03-28

**Authors:** Edmund Howe

**Affiliations:** Department of Psychiatry, Uniformed Services University of the Health Sciences, Bethesda, MD, United States

**Keywords:** treatment, trust, challenges, validating, values

## Abstract

Many patients have lasting disorders due, for example, to excessive and chronic childhood stress. For these patients, certain psychotherapeutic approaches may be maximally effective, and this may be universally the case. This piece is intended to give providers optimal tools for reaching and helping these patients who, otherwise, may remain among those worst off. These interventions should enhance patients’ trust, the quintessential precondition for enabling these patients to change. Specific interventions discussed include anticipating ambiguity and clarifying this before ambiguity occurs, therapists indicating that they will support patients’ and families’ wants over their own views, feeling and disclosing their emotions, validating patients’ anger, laughing, going beyond usual limits, explaining why, asking before doing, discussing religion and ethics, and informing whenever this could be beneficial.

## 1 Introduction

Psychotherapy can change people’s lives. It can change the quality of their life, from dreading every tomorrow to enjoying every day. Some clinical practices bring about these changes more than others, though all may need to be adapted to patients’ different cultural values and beliefs. One review of leading psychological interventions found, for example, that among many interventions, 10 were most likely to be effective (1). These included what these authors call “affirmation and validation”, paradoxical interventions, behavioral activation, and cognitive restructuring (2, 3). Many of these interventions, as others not listed here, are used most often for specific disorders. Many patients have, however, less specific, longer-lasting disorders, as those deeply affected, for instance, by chronic childhood stress. For these patients, more general approaches may be or are more effective, and this may universally be the case. As the above authors state, “Although some psychotherapies may make better marriages with some mental health disorders, the repeated Dodo Bird conclusion in general as well as for most disorders indicates that bona fide psychotherapies produce similar outcomes, once the researchers’ allegiance effect is identified and controlled … optimal outcomes come about when empathic therapists collaboratively create an optimal relationship with an active client on the basis of the client’s personality, culture, and preferences. Clinicians strive then to offer a therapy that fits or resonates with the patient’s characteristics, proclivities, and worldviews—in addition to diagnosis” [(4), at 1890–1, (5)].

This issue of *Frontiers in Psychiatry is* dedicated to helping patients, especially those with emotional needs who are worst off, to acquire equal access to treatment. In this piece, I hope to enhance providers’ ability to accomplish this with all patients. I will refer to all medical personnel who may help these patients as “providers” throughout this piece since they may not only be doctors but also nurses and other providers. I will discuss several interventions, ranging from those offered by experts to those supported by empirical studies. Some measures may already be known to most providers, but even these providers may still find suggestions that add to their practices. Most interventions suggested should increase patients’ trust, and this effect in turn may enhance patients’ capacity to respond to whatever approaches providers are using (6). I will do this in three main sections: challenges; interventions, both general and specific; and controversies. For each suggestion discussed, I will provide illustrative case examples. I will also, after each, present in a sentence or two the core suggestion that providers may take from the prior discussion. I am hopeful that the approaches suggested may help providers reach particularly those patients who are worst off. It is these patients who most likely may need them.

Providers seeing these patients regardless may conclude prematurely that they are not able to help these patients. Providers have seen these patients, and these patients still may, for example, just want a place to stay or are primarily seeking attention. These patients may be discharged to their homes with no plans for follow-up. These patients may, though, have hidden and more severe underlying problems and, thus, need more help than others.

A real-life example here is “Tina”. She was diagnosed as having borderline personality disorder, a disorder often applied to patients more difficult to treat (7, 8). Tina had, from the time of her early teens, spent most of seven “long” years in psychiatric hospitals, “drugged up to her eyeballs”, she says, in psychiatric meds. [(7), at 16)]. Then, however, she met a therapist who believed that what Tina most needed was understanding. This insight began “a huge turning point” in Tina’s life. She then did well, and if she had not had this help, she later said, she would have, she believes, been “medicated all the time”, and her providers would have continued to see her as “more than anything else” an untreatable, difficult, “attention seeker”. [(7), at 17, (9)].

This piece is mostly intended to help providers treat patients like Tina.

## 2 Challenges

Five challenges, I believe, prevail: to help patients feel safe, gain their trust, not judge them, engage their feelings, and show feelings.

### 2.1 Helping patients feel safe

Providers must help their patients feel safe. This is an absolute priority because if patients feel fear, this alone may preclude their capacity to respond to therapy. Many patients feel fear when they start therapy. They may fear, for instance, that if they are honest about themselves, their providers will disrespect and judge them, and then they will feel shame. Thus, patients may withhold information initially and always avoid this, or they may, in some way, verbally fight. Providers must anticipate this. If patients do fight to protect themselves, providers must address their underlying needs at once, not merely or even primarily set boundaries.

The example I will use as a paradigm for this situation involves fear that patients may feel whenever they perceive something their provider does is ambiguous. Patients who feel fearful or anxious see ambiguity more readily than others (10–12). They are more likely also to, then, infer that the meaning is most negative toward themselves even when this is not at all the meaning their provider intended (13–15). An example is a patient who had to reside in the hospital to receive daily treatments there necessary for her to survive. Her family was large, and family members visited her in shifts throughout the days and early evenings. This worked ideally for all. They found meaning and joy in this arrangement. Then, however, a provider updated in medical ethics feared that she might not know that if she chose to, she could decline her life-sustaining treatments. Thus, the provider told her this. She, however, saw him as meaning to suggest to her that she should end her life in this way—that if she did this and died, her family members could then go on with their lives without feeling that they had to visit her. She declined all treatments the next day and died shortly thereafter.

What this provider said was ambiguous. He had meant to just be sure that she knew. She read into what he said and what he did not intend. A possible remedy that providers may want to consider is anticipating ambiguity in what they say and then alerting their patients to this ambiguity before they speak. They can tell them that this alternative meaning, though present, is not what they intend, but they do not know how to say what they want to say in any other way.

*Anticipate ambiguity. Clarify this before speaking.*

### 2.2 Trust: the pre-condition for patients to do better

Helping patients feel safe is a necessary task. Earning their trust is a close second (16–18). How providers may best do this may be paradoxical. Providers knowing the importance of trust sometimes directly urge their patients to trust them. Why else, they might say, would they become providers, if they did not want to do for their patients what is best for them? This urging may, however, be counter-productive. It may evoke guilt, not trust, because patients, like all people, may simply not be able to *will* themselves to feel trust. Trust must come about on its own.

What, then, can providers do instead? What they can do is somewhat analogous to their forewarning their patients of ambiguity. They can advise their patients to *not* trust them—to not trust them until and unless this trust comes about naturally. This newfound trust may occur, providers may suggest, when the patient feels for whatever reason that the provider has earned this trust.

Providers can go beyond this. Providers can imagine the needs that patients could have that may even be not known to the patients and then pursue ways of meeting these needs. As an example, when providers first anticipate that there could be an ethical conflict between patients or their families, on the one hand, and medical staff, on the other hand, providers may take the initiative to tell these patients and their families that if this conflict surfaces, providers will support them in every way possible. Providers may, for instance, lead patients in bringing an appeal, or if there is no avenue for this, seek to create one. Most critically, providers can indicate that if these parties want providers to do this, providers will seek to support these parties maximally, regardless of what providers believe. Providers may wholly disagree with what these parties want but still pursue with them what they want, *absolutely*, because it is what these parties want that alone counts.

There are two possible downsides. First, providers saying this, especially early on, may evoke these parties’ fear, and this conflict may not later arise. As an example, the ethical conflict the provider anticipates may involve futility. The patient may, for instance, be dying from heart or liver failure but also have kidney failure. Renal dialysis, started at even this late time, may prolong this patient’s life for a few weeks. The patient and family may want this. They may cherish this longer time with each other. The medical staff, though, may see their starting dialysis at this time as not life-prolonging but death-prolonging and futile. Staff may be concerned also that their initiating dialysis at this time would deprive other patients of the time and care that should be given to them. Thus, the staff may refuse to initiate dialysis and, legally, be able to do this. The staff may also have their hospital’s explicit permission to do this. Providers calling this later possible conflict to patients’ and families’ attention may cause them unnecessary worry. Second, providers taking on this role on behalf of these parties may pit them against their own staff. These providers will have to be working with them later.

What, if any, might be the best limits to providers taking the above initiative? A threshold question that providers might want to ask themselves is what these patients would want if they had at their bedside 1) a specialist in their medical illness, 2) a lawyer, and 3) an ethicist and were wealthy enough to pursue in court what, with these three people’s input, they have determined they want. This might be, for example, their having kidney dialysis, as in the situation just discussed. Those with assets can now seek this remedy. If those without these assets could not, this would violate their having equal access to treatment and thus might especially warrant providers’ making this offer and intervention.

*Support patients’ and families’ needs maximally. Tell them you will.*

### 2.3 Our need, asymptotically, to not judge

A fault we all have and cannot, I believe, ever fully transcend is our proclivity to judge others. This includes judging our patients (19, 20). This tendency, like people gaining trust, may be outside our control. If we do judge our patients, this may be evident to them. Consciously or unconsciously, they may pick up on even non-verbal responses we do not know we are showing or even having. An example is our unknowingly raising an eyebrow (21). If this occurs, this may wholly negate our doing effective therapy by leaving patients feeling unsafe and losing their trust.

Why might we judge even when we strive not to? This may be in part because we may respond immediately to what we experience with felt judgments. This immediacy may have, through our evolution, helped enable us to survive. We may though, just moments later, find our minds flooded with logical reasons that support whatever we have felt. Nevertheless, these reasons may be spurious and unbalanced, and we may not know this (22–24). Unknowingly, we may then cherry-pick our reasons and have become more certain that our judgment is sound, not biased (25, 26).

A first step in combatting this risk of judging our patients is to check out objective grounds to try to see better whether our initial feelings and logic should hold (27). There are two other steps we can also consider taking. The first is to seek to get to know patients toward whom we feel bias better. When we know people better, our feelings toward them often shift. We may value them more as if we might our child or a close friend and see them more clearly as they are but less judgmentally.

When, if ever, should we *not* do this, but let our initial judgments stand, as they are? A test case example here might be parents who oppose providers giving their child pain meds because they believe these meds are unnatural and thus this would violate God’s will. When this occurred, I suggested that this child’s providers relieve this child’s pain at once, which they did. Nevertheless, one can see these parents’ views in a way that is not judgmental. They may want their child to have eternal life. More generally, providers may not be able to have it “both ways”: they may not be able to treat others optimally while at the same time continuing to judge them. Providers’ judging, always, may “show through”.

A second possible means of reducing our judging is to try to see things through our patients’ eyes to the degree that we can. People making this effort may successfully overcome their previous view of their patients as “other”. There may be no limits here. Some have, by this means, come to feel both alike and bonded, for example, with patients who are homeless. I have experienced this, as I will later describe in more detail.

Here, again, there are downsides. These may be so subtle that they often are not known to us. We may feel greater empathy toward these patients as a result of our knowing them better, but, feeling this greater empathy, we may inadvertently do harm by distancing ourselves from these patients. This may be an unconscious response geared to lessen our own pain. We may, worse, again not intentionally respond then with pity (28). Still, the gains achieved from our making these efforts, relative to these harms, may be immense.

We are particularly prone to judging others, finally, for not taking responsibilities that we believe they can and should take. An example here, one perhaps especially painful to imagine, is providers deciding what they will do when they foresee that the genetic testing they may do on children and their parents may reveal that a father is not biologically related to his, her or their child (29–31). Providers encountering this situation may tell themselves that if these findings break up these families, the mothers get what they deserve because their irresponsibility caused this. This is the kind of judgment that providers should eschew.

*Discern even tinges of judgments. Seek to undo them.*

### 2.4 Feelings: ask about these as well as patients’ thoughts

Feelings and thoughts may oppose each other. Thoughts may correct feelings, as we have seen when discussing our judgments. Feelings may guide us to see realities that we, using logic alone, may have missed. Providers may, also, pursue with their patients their gaining new insights from their feelings. This may be particularly likely since cognitive therapies tend now to so much predominate. When patients are re-experiencing terrifying feelings they once felt based on reality, they can learn to remind themselves, for example, that in their present situation, these feelings no longer reflect the threatening situation they once were in.

Pursuing ways in which patients can change painful feelings more directly may still, though, also be an important intervention. They may, too, share more troubling memories after each initial feeling that they share.

An approach called accelerated resolution therapy (ART) is, for instance, an example. ART involves patients envisioning the beginning of a past traumatic event and then re-directing in their mind’s eye what occurs from there so that, in their newly imagined vision, they re-see what occurred in a way that is positively resolved (32). One patient, for example, as a teen had been assaulted. She then imagined in her mind’s eye this person disintegrating such that his tiny parts fell into the soil and fertilized beautiful flowers that she then gave to children. She reported, immediately after this, feeling better.

Providers, as a second example, may ask patients having a painful feeling to re-create it in a way that is only mildly painful, and then while patients hold on to this feeling, talk them back through their life to see what other experiences, if any, come into their mind. This technique is called an affect bridge. Here, an example is a patient who was in love with a man she wanted to marry but felt discomfort when they began to become physically involved. She did not know why. I asked her to re-create this feeling in her mind and then guided her verbally while she was holding on to this feeling, back through the years of her life. Suddenly she shrieked, not in fright but in astonishment. “My boyfriend’s eyes are just like those of a boy who bullied me during grade school”, she said. Seeing this similarity for the first time, her negative response to her boyfriend then decreased, and they got married. This response is known as an “Aha!” reaction. Further, if patients have this response and providers tell them that when this happens their painful feelings may decrease, their knowing this may make this more likely to occur.

*Elicit patients’ feelings as well as their thoughts.*

### 2.5 Consider sharing your own feelings

I have marveled and been struck again and again by how different providers experience different feelings toward the same patient. Sadly, on occasion, providers may, for instance, share behind closed doors that they dislike a patient, but one provider may greatly care for this same patient. How might this occur?

Providers assessing this situation may well regard this outcome as resulting from patient-initiated “splitting”. They may see this patient as pitting this one provider against all the others—consciously or unconsciously *manipulating* these providers, much as some believe children may seek to split up their parents’ views regarding something they want.

A different possibility and alternative explanation not considered so frequently, perhaps, is that the one provider still caring for this patient may be simply more emotionally gifted and relate to this patient and generally to people better than others. This possibility has far-reaching clinical implications. It may make the difference between patients doing badly or well. Two examples will illustrate this: the first involves parents who have just given birth to a child who is stillborn or who will die soon or foreseeably within days (33–35). These parents may feel at this time emotionally devastated. They may want above all else to avoid even thinking about, much less seeing, their deceased or dying child. Nevertheless, some parents in this situation can do better and remarkably so if they then can, notwithstanding their initially being in this state, somehow move themselves to see, hold, and bathe their child. This experience may transform their unbearable grief and even bitterness into what in their words may become a “beautiful” memory. They may change from believing that they would never again seek to have a child to wanting to have another or many children.

Providers knowing of this transformative possibility may believe that they should at least inform these parents of this possibility. Nevertheless, they may fear that no matter how they do this, these parents may feel enraged, thinking, “How could our provider suggest this to us at this time?” This may be an instance in which only the most emotionally gifted and psycho-socially skilled providers could inform these parents of this successfully without triggering parents’ hurt and even rage.

A second example involves parents who have a child in grade school or above who is dying but who do not want their child to know this (36–38). They may have, again, understandable reasons. They may, for instance, not want to frighten their child. Thus, they may withhold this information, and their child’s providers also may not tell the child this to respect these parents’ decisions. This child as a result, though, may die feeling terribly isolated and all alone.

Some providers who have personally encountered this situation say that most often or at least often, these children already know that they are dying and, even if they do not, they do much better if they can share with their parents what they are feeling as these children die. Nevertheless, again, only the most emotionally gifted and emotionally skilled providers may be able to persuade these parents to inform their children that they are dying without evoking only the parents’ anger. Only these providers may be able to convey successfully to these parents that their children may suffer much less if their parents tell them that they are dying and that these children, *as these parents*, may find this sharing most meaningful.

Some providers relative to others, then, lack these skills. Nevertheless, they may still have to do the best they can with the skills that they have. How might they best do this? I would suggest here that what providers may best do is to identify the feelings *they* have as opposed to hiding these feelings and letting these parents see the grief and pain that providers also share, making clear that they know that their pain is, as it were, infinitely less. Providers may view sharing what they feel is “professionally” prohibited. Providers may have been taught during their training that they must at all cost maintain emotional distance and not overly respond to their feelings, both to be able to function satisfactorily as providers and to not lose their objectivity due to their becoming too attached to their patients. These absolutes could preclude providers, for example, from ever crying in the presence of a patient (39).

Nevertheless, if and when providers feel, but do not show this, patients and parents may both feel more alone and, as a consequence, feel less able to bear what they find unbearable. Patients and family members have said that when they were dying or a loved one was dying and a provider also felt sad and showed this, the provider’s crying helped make their loss more bearable.

This same consideration arises also for providers who ethically consult. Then, they may exceptionally focus on which moral principles they should prioritize to help bring about the optimal ethical result. This effort principally involves abstract analysis. This effort may, however, take them further from recognizing their own feelings and deciding then whether to share these with patients—and parents. Their struggle to identify which among competing moral values should prevail may also distract providers from seeing how their patients are feeling so that they can then respond to these patients’ felt needs, which—I would suggest—*always* should take precedence. This is because any feelings that the patients have are likely to interfere with their being able to best hear and understand what their providers are saying to them so that they can then respond optimally.

It may be as some have said that there is a “hidden curriculum” at some medical schools that leaves medical students less fully attuned to their patients’ feelings over time. If true, it may be also that when providers encounter ethical conflicts, there is a similar “hidden ethics curriculum” taking place as well. This curriculum might move providers as they go through their ethics consultation training to focus progressively more and more on ethical analysis and less on patients’ mutually exclusive emotional needs. This may be exemplified by what occurred when the patient related above misinterpreted her provider’s ambiguous information and chose as a result to die the next day. He may have focused almost exclusively on the ethical importance of his enhancing her autonomy. He may have simply missed the more subtle, non-verbal manifestations of her hurting if, while he was speaking, she imagined that he might be the right—that she should give up her life for the sake of her family members.

*Feel. And do not be afraid to then show this*.

## 3 Interventions likely to be optimal

### 3.1 Interventions

#### 3.1.1 General

In most psychotherapeutic endeavors, compassion and trust must be conveyed for clinical interventions to be maximally successful. The first moments of the first session may be exceptionally important (40). An example illustrating how even the smallest interaction may be important at this initial meeting involves how providers may best escort new patients from the waiting room to the treatment room after first greeting them. Should the provider lead, follow, or walk side-by-side? Providers may see this question as excessively conscientious. It is, however, not only important in its own right. It also illustrates paradigmatically how providers may best pay attention to all aspects of their interventions, such as, also, to consider both “the angle” and how far from their patients they should sit. All the interventions I will subsequently discuss here have a small but similarly important effect especially when considered together.

##### 3.1.1.1 Validating patients always takes precedence—even and especially when they verbally “fight”!

Patients may fear that their providers will judge them as we have discussed. This fear may exist in regard to every word their providers say. Providers can help alleviate the likelihood and intensity of this fear by initially validating at least some aspect of what patients say. This conveys at least that providers are attempting to understand, as we can consider and imagine was so precious to Tina. Validation can *always* be genuine. There cannot *not* be some sound or valid rationale that underlies all patients’ thinking no matter how illogical or irrational their conclusions may first appear. Some providers are, however, much better at discerning these underlying rationales. Those providers lacking this skill—this application of their imagination—must recognize this and do what they can as we have considered already, analogously when discussing providers who have greater psycho-social expertise relative to others. Here, providers may best acknowledge, again and again, if necessary, how much they want to understand what is most important to their patients. They must do this until they feel they have a sense of why their patients want what they do.

The example I will use here involves patients who, in one way or another, non-verbally “fight” in a non-dangerous way. An initial response often urged is to immediately set a firm boundary—to be sure that the patient knows what the provider sees as an absolute limit to what and how the patient can respond. “I will not tolerate this”, the provider might say.

This demonstrates, of course, unequivocally, that the provider has “the power”. This may, as well, be emotionally infantilizing for the patient. This may then ever thereafter limit the closeness each may feel for the other.

An alternate approach is for providers to not respond in this way, but rather, initially, to imagine that these patients are likely responding to something important to them. They may, for example, have fear regarding their illness. This “fighting” may come about, then, from their unconsciously defending themselves psychologically from this fear. Possibly, this fear may be more painful than anger to experience. Providers, then maintaining emotional control within themselves, may empathically inquire: “I may have said something insensitive. If I did, I’m very sorry. And if I did say something insensitive, if you would be willing to, could you tell me, so I can meet your needs better?” (39, 41)

This way of responding hopefully expresses unequivocally that the provider does not want to fight back or silence the patient by showing who has the power. These patients may well know that they responded in a hostile way and then may appreciate the provider’s effort to better understand. This provider’s extra effort may not only reset their exchange but also re-establish a positive relationship that then continues.

Providers’ memory of patients’ hostility may also linger within them. They then have more work to do to reduce, ideally completely, their initial, possibly retaliatory reaction. Approaches they can use to pursue this end roughly mirror those discussed above for their undoing patient-disfavoring moral judgments. As an example here, we may again consider the couple we discussed above who sought to withhold pain-relieving meds from their child because they thought that these meds were unnatural and that thus their giving these meds to their child would go against God’s will. Providers could then validate these parents’ view even as they acknowledge that they must go against it, always, also, of course explaining why they are going against what these parents want, which is in this case to spare their child’s feeling pain (42).

I experienced what may be a surprising and extraordinary response to providers’ validating others’ views. I was an ethics consultant when a conflict arose between the family and the staff treating a middle-aged woman who was then in a coma. She had remained totally unresponsive to every intervention her providers thought could be even possibly beneficial. She had remained in an ICU in this unresponsive state for weeks. At the staff’s request, I met her family, together, to discuss this. There were five family members who were the mother’s adult children. All five of both family and staff sat opposite each other on a long table. I sat at its end. The sole question raised and the one that the staff wanted to discuss was whether it was time “to respect this mother’s dignity” by discontinuing her treatments and allowing her to die. The family objected. “But we—you”, they responded in unison, “*still* don’t know what is wrong”.

I then concurred with them. “The family is right,” I echoed. “We *don’t* know what is wrong.” This uncertainty, I could imagine, as did the family, increased the possibility that their mother could recover, although the chances were slim. The staff looked at me with surprise. I was supporting it seemed the family’s view, not theirs. I was, but I was more than this, seeking primarily to validate the legitimacy of their contention.

There was then silence. Then, suddenly, the leader of this family said, “Maybe the staff *is* right. Mom could get better, but she hasn’t. Maybe we should accept this and let her die with dignity.” All the family then in short order agreed with their leader, though they had held the opposite view just moments earlier. Their mother’s treatment then was withdrawn. She ironically fully recovered and left the hospital, walking, 2 weeks later.

Why did the family’s view change? I would suggest that this family’s decision to go “the other way” stemmed, at least in part, from my having validated their point of view. Just one other person’s support may in many cases have this same effect. This particularly may be the case with patients and families when the person who supports them is a staff member. With this support, people, psychologically, no longer have to put so much or all of their effort into defending their challenged point of view. Rather, they can, to a greater degree, then, let this seeming necessity go and look at the problem confronting them more objectively, possibly coming out as they do here, the other way.

*See and acknowledge what is compellingly sound in all that patients and families say*.

##### 3.1.1.2 Highly value meaning even when there is none

Many know of Viktor Frankl (43–46). He, after losing family members and surviving in a concentration camp, came to believe that those who like him had survived this experience may have and have had in common a sense that they still had meaning in their lives. This possibility has been supported much more recently by the gains that therapists have found in empirical studies on positive psychology (47). Based on these findings, some experts have suggested that as opposed to therapists focusing mostly or entirely on reducing so-called negative symptoms such as anxiety and depression, they should focus more, instead, on increasing patients’ capacity to experience positive feelings, such as, principally, to be able to feel meaning in their lives and to be able to enjoy humor (48, 49). We shall consider humor subsequently.

Providers have focused on meaning in the past, particularly with patients who are dying. A most profound question sometimes emerging in this context is how patients can continue to find meaning in their lives when medically they have no way of living longer. These patients may find additional or new meaning in leaving their loved ones as their grandchildren able to retain positive memories of them even as they are dying. Providers often urge them also to find meaning in what they have passed on to others.

I am struck here by what may be left out of the above conversations. This is because, at these times, when patients are dying, they may want more than anything else to be able to discuss not their coming death nor what this means but everyday matters, as if they were not dying. I recall one patient, for example, with whom we discussed, as he died, nothing other than art. We would pursue this each time we met as though his dying was not an issue. I recall another friend whose skin was yellow due to jaundice. He attended professional meetings regularly just as he always had until he died. With still another person, we discussed how couples otherwise getting on well sometimes often both drink on Saturday nights and then virtually every weekend get into fights. This person, a patient, expressed how particularly priceless to him these discussions of everyday happenings were since they so distracted him.

Some patients find no meaning in their lives. I think here of a patient whose sole meaning in life had been to care for his wife who had a fatal disease and was slowly dying. He then himself had a rapidly fatal disease that would end his life before his wife would die. He felt despair. His life goal no longer existed. What can providers do then? I could be with him, sometimes just sitting. He told me he preferred me there as opposed to my being away. He shared with me then that he had asked a friend to bring him a gun to end his life, but his friend would not. When there is or may be no meaning for a patient, just being with another person who knows this may provide meaning—the meaning of just being *with.* This possibly, for many, is in actually *every* context, what life has most to offer.

Providers considering meaning with dying patients may be more important than any other interaction, however, for an additional, less obvious reason. When we so converse, we are more clearly than usual, *with* our patients, simply other persons who like them share the same destiny. Both will die, although, at the time, one is dying while one is not. Both have this in common then, although, at the time, each is in a different role. This too may at the deepest level help patients not feel so alone.

*Join dying patients. Whether they feel meaning or they do not.*

##### 3.1.1.3 Humor—best when the one laughs at the provider!

Frankl says that humor is most important in therapy. It distracts, he says, as we have just considered discussions of everyday life, and more than this, puts pain into deeper perspectives. To initiate humor is, however, risky. Patients are hurting. Providers offering humor at this time, then, may seem to patients insensitive, like asking parents whose infant has died or is dying if they would then want to see, hold, and bathe their baby. There may, though, be means of threading this seemingly impossible-to-thread needle. Providers may *ask*. They may say, for instance, “I find myself wanting to say to you now something that involves humor, but I fear that humor may be the last thing you would want at this time What do you think? Shall I share with you what I thought of saying?”

Questions often arise that similarly may help or harm. A most common example here is how providers should speak of patients’ pain. Should they risk “overstating this”, as patients experience this pain, or risk, referring to this pain with words that may call it less than it is? The “price” from this first route, providers saying “pain” when this may be to a patient little more than discomfort, is, in the view of some, that this may reinforce and thus increase this pain. It may even, they say, have a placebo-like effect and create pain where there was only discomfort by suggesting this. Nevertheless, providers using “lesser words”, like “discomfort”, may risk connoting to patients that their provider is not taking the degree to which they report their pain seriously.

Possibly the best resolution between these two risk-laden options is to choose not on the basis of the greater risk or best net effect but to base this decision on a different determination altogether—namely, on what may most strengthen or harm the relationship. Here, overstating this pain, like sharing humor, may in spite of their risks increase the caring between the two.

What is the highest goal providers might strive for with humor? I would suggest that this is for patients and providers to become so comfortable with each other that the patient can poke fun at the provider. I recall such an instance. A patient had been most stricken with medical issues. Due to his many exceptional problems, he often needed urgent appointments, such as to reduce suddenly occurring pain. Nevertheless, due to a busy schedule, he was sometimes told he would have to wait for a substantial time. I had once intervened to try to get him an earlier appointment and succeeded. Some would oppose my doing this. They might hold that providers should not use their medical identity to seek special privileges for their patients, but rather, if the system needs to change, to try to change it. I do not.

In any case, this same need again arose, and again I made this offer, to intervene on this patient’s behalf, so that he could then get an earlier appointment and earlier severe pain relief. He said in response, “No thanks, not now. I have one other call I can make that could result in my having an appointment sooner. Then,” he joked, “I’ll *sik* you on them!” adding that he hoped his joking *at me* in this way I understood as conveying the warmth he intended.

*Laugh with patients. They and providers need this*.

##### 3.1.1.4 Providers going beyond their usual limits

Providers not uncommonly self-sacrifice at least to small degrees when this is necessary to benefit their patients (50–52). This goes with the nature of the medical profession. Such sacrifices may be what patients need. Nevertheless, providers’ sacrifices may also go beyond this, and when providers do this, patients’ trust may increase immensely. When, if ever, providers should do this is, however, subject to disagreement.

A paradigmatic example illustrating this is patients who have insomnia and need a sleep med to be able to sleep. Providers may see one night’s sleep as not worth their significantly self-sacrificing. Nevertheless, patients may respond to this problem in highly different ways. They may find lying awake in bed all night, for example, excruciating. Thus, they may suddenly on a Friday night first notice that they are out of sleep meds and then call their provider if they can in a panic, asking for help. Local pharmacies may then be closed. Providers may though accompany these patients at this late hour to find a pharmacy, somewhere, open all night and then find a way to enable these patients to get this medication and to sleep. This may require this provider to even go in person to this pharmacy because this sleep med is a controlled substance and the pharmacy may not be able to give it out in any other way.

Some providers encourage patients to call them at any time if they have such an urgent need, though they also provide a backup resource in case their patients cannot reach them. This possible full-time access has saved patients’ lives. Patients calling 911 or now 988 when patients are suicidal may not, for one reason or another, suffice. John Gunderson, a psychiatrist and expert on treating patients with borderline personality disorder, made himself available to his patients “24/7” and described how he would respond to patients when they called him because they felt suicidal at an annual psychiatry meeting. He would urge them to go to an emergency room immediately. He was not, he said with a grin, his “usually charming self” on the phone at these times (53, 54).

The gains to patients from providers sacrificing their own interests, as Gunderson did, to help patients may be, as I said, most impactful, possibly even, saving patients’ lives also in other ways. One patient, for example, refused to take anti-cancer meds, though taking them, his providers told him, would likely extend his life for more than a decade.

One provider then copied several articles that documented this claim and after work spent several hours showing these and discussing each with this patient. He finally “gave in”. He took these meds and responded as predicted. This provider wondered whether his effort was too excessive. I would wager that it was not these articles that changed this patient’s mind but the caring commitment this provider showed to him. That is, the change agent, I suspect, was this provider doing this for him to this extent.

What should providers say if patients ask for help but providers will not go for them this second mile? There may be many reasons, of course, for this. Chief among these may be, for example, the law. Patients may, for instance, want off-label medications, but providers may see this as perhaps beneficial for these patients, but leaving themselves too vulnerable to being sued or even being sued if these patients have complications. Should providers, then, acknowledge to these patients that they could prescribe them and that other prescribers might, but that personally they are emotionally more risk-averse and unwilling to prescribe what the patient wants and even may need for this reason?

When providers are more fearful than others and disclose this, patients’ responses may be more understanding of this and positive than providers may expect. They may understand that their providers are like them and say something like, “*I’m* sorry. Of *course*, I wouldn’t want you to have something bad happen!”

*Consider going the second mile.*

#### 3.1.2 More specific interventions likely also to globally be optimal

There are, in addition to the above more general approaches, several more specific interventions especially likely to be optimal for most patients worldwide. This may be the case notwithstanding the psychiatric disorders these patients have. This is because these approaches each are responsive to patients’ universal proclivities, such as their wanting to understand why their providers are doing what they do, as opposed to patients complying without knowing why. Patients widely, as a second example, prefer having choices. The outcomes may be much the same, but the effects of subtle differences such as those described here may over the long run be more successful.

##### 3.1.2.1 Explain *why*: to teach, respect, and more “equalize”

Therapists should always consider explaining why they will do what they will do before they proceed and “just” do it. Having explained, they can then ask patients whether they want them to proceed, and patients who are better informed at this time may then give different answers. Explaining “why”, in addition to better informing patients, grants them more respect and implicitly renders them then or connotes them more as “equals”. To the degree that patients know more, they, of course, *are* more equal. This practice also may enhance the degree to which patients and providers work together. These additional explanations may finally give patients more hope.

I recall a patient who feared leaving his house. He had had staggering personal losses throughout recent years, and the effect of this continuing stress had seemed to affect his functioning greatly. He had, for example, extreme agoraphobia. I described to him how gradual desensitization worked, emphasizing that we could start with as minimal a new slightly anxious activity as he was willing to bear. He had a porch. “Could you go a step out, I asked and then stay there, even if only for a few seconds?” He could, he said. He shared then later that his understanding of *why* he must take the initiative to do this as opposed to his just waiting for his fear to wholly wane as he had, enabled him to accomplish this breakthrough. In time, he was able to fly to his child’s college and attend his graduation. He also shared after this that my explaining to him in detail why this might help meant to him that we were sharing this task *together*, as opposed to the responsibility for its success or failure being his alone.

*Explain, even when and what you, the provider, do not know.*

##### 3.1.2.2 *Ask*. Respecting patients and at this same time lessening the risk of their being oppositional

All requests can be converted to questions (9). “I think you should do this”, can be converted, for example, to “Would you be open to doing this?” A provider’s asking this question converts this request into a question. A provider also can ask such questions, “Would you like me to tell you *why* I believe this could be helpful?” Providers sharing this can convey additional hope. Both these questions, of course, explain the why of the intervention proposed, and the change in sentence structure to questions may result in the patient accepting the request as opposed to partially or fully resisting it.

Providers asking as opposed to telling—or even just suggesting—lessens the risk of eliciting within patients an automatic, unintended, oppositional reaction. Asking them places them in the driver’s seat. Questioning allows them to decide what they want, regardless of what therapists believe is best. This also is more empowering.

This does not mean, however, that providers should keep any view that they believe might be helpful to themselves to avoid triggering an oppositional reaction. Rather, caring for patients should mean sharing with them any and all considerations that they believe patients may want to know and could find helpful. Providers asking them if they would want their provider to share this additional information may also give them this additional choice.

Patients may ask therapists, as a first paradigmatic example here, for instance, “What would you do in my situation?” Providers may feel clear about how *they* would answer this question but believe that they should not disclose this because they are not their patients and have not been living their lives. Thus, they may see their saying what *they* would do as irresponsible. Providers may feel this way also because they know that just a few decades ago providers tended to believe that they knew better than patients what was best for them and often acted alone on the basis of this presupposition (55). Then, they might not tell patients that they had cancer, for instance, because they believed that these patients might kill themselves. They came to see this and other paternalistic acts as empirically mistaken as well as unethical. Their remedy was to remain neutral, and this resolve has persisted. Some providers share with patients now for this reason, only and exclusively, factual information.

A preferable approach then may be for providers to take every opportunity they can to give patients, after asking, information that they believe, plausibly, could be helpful. Providers can imagine and then can offer to share these risks of their saying what they would do before speaking. If they say what they would do, the main risk is patients going with or going against what their provider would have done and then regretting this. “I should have done what my provider would have done or not have done this just because this is what my provider said,” and then they may blame themselves later. There is here, though, a much stronger rationale for providers to say what they would do notwithstanding these risks. Namely, providers not sharing this may leave patients feeling emotionally abandoned by their providers. Patients’ feelings and the relationship again as always should be the providers’ chief priority and concern.

Similar concerns may arise when patients or families ask providers whether patients should have cardiopulmonary resuscitation (CPR) or request a do-not-resuscitate (DNR) order. Providers often have very strong views that they should not further provide futile treatments. They may in this instance also not want to risk breaking patients’ ribs during CPR attempts only to have these patients die shortly thereafter. Thus, their tendency may be to respond with a “top-down” answer, sharing strongly what they feel is best. Providers doing this risk implying that they believe they know what is best for patients more than patients do. They may in this instance. They have medical knowledge and experience that their patients lack. This still, though, may not be sufficient to equip them to know what here is best for a patient.

What could providers do instead that may not leave the patient feeling emotionally abandoned? They could ask, “Would you like for us to together discuss this?” They could continue, “This answer, what is best for *you*, may depend on concerns most precious to you but that through discussion we may want to newly discover”. This discussion may, of course, take more time. Some patients in medicine inevitably take and need more of the providers’ time than others. The above patients may be among them.

A final consideration here is whether providers asking patients whether they would want to discuss this together is too coercive (56). Could the patients say, “No thanks”, without feeling uncomfortable? Providers could, to help allay this risk, share with these patients the bind they imagine patients are in when they ask them this question. “We could discuss this for the reasons I’ve said”, providers might say, “but I fear that my just asking you this may make it hard for you to say, ‘No’. Nevertheless, whether you would want to discuss this depends wholly on *you*. Some patients discussing whether they would want CPR or a DNR order find this analysis complicating matters and thus being more painful. Others find that this helps. Which, then, would you prefer?”

The risk of providers inadvertently evoking an oppositional reaction is commonplace and universally present. Some patients have but hide their angry response. This may be harmful to them. This stress, unexpressed, may, for example, have a physically negative effect. Others though, as we have addressed, may non-physically fight, such as by having a clear edge in their voice. This oppositional response, warranted or not, may disrupt the relationship then and thereafter.

*Ask. Re-phrase “top-down” thoughts as questions.*

##### 3.1.2.3 Self-disclose, though risky

Self-disclosing is no doubt risky. Patients, first, may not want to know or hear anything about their provider. They may think, “This therapy is about *me*, not them. Don’t they know this?” Worse, and second, what providers divulge may diminish patients’ confidence in their providers’ competence. This may though too work the other way and benefit patients in ways that providers do not anticipate (57–59). One provider’s disclosing that he was divorced evoked in his patient, for instance, an “Aha” response. This patient said to himself then, and only then, “Maybe I’m all right as I am. If my provider is divorced and doing this well, maybe, though I’m divorced, I’m okay too”. He stopped therapy shortly after this and his life went well.

This example illustrates how providers’ sharing information about themselves may benefit patients. It may particularly reduce patients’ shame and guilt by modeling for the patient that their provider is not perfect either (60, 61). I have at times shared my own imperfections for this reason. I told one patient whom I had just called, for instance, that though I had just called him, I had misplaced his phone number while talking with him on the phone. I then asked him to give me the number again. This may have lessened a top-down assumption he had regarding me and our relationship. He was handy with a hammer. He then sought to advise me. “Do you know how to best hammer in a nail?” he asked. “No,” I said. “You tap it first,” he said. “Then you can hammer it in better.”

*Self-disclose. Delicately. Decide where to go then from there.*

##### 3.1.2.4 Reframe: what is half-empty always is half-full

Patients have any number of ways in which they can most ingeniously put themselves down. Cognitive therapy, of course, seeks to teach them how to recognize when they are doing this to themselves and how then they can more objectively reframe what they tell themselves. Providers can model this by putting whatever patients say negatively regarding themselves in a better but still sound light. That is, all glasses, half-empty are also half-full. This models for them what they can do also on their own.

I think here as an example of a father who felt deep despair when his daughter told him that she was getting a divorce. “I should have warned her,” he said. “Her husband was penniless when she met him. I should have warned her that this wouldn’t work.” I told him then that I *totally* disagreed with him. I may say this using exactly these words when I know the patient will take this as my intending to share a caring endeavor and as my wanting them to particularly attend to what I will next say. I said to him then, “I think that instead you should be proud, *very* proud of yourself. By not opposing your daughter’s marriage or even questioning it on financial grounds, you gave her the, perhaps, most valuable lesson a parent can give to their child to value persons for who and how they are, not for their money.” He was staggered. He agreed with my view upon hearing it. He reframed at this moment how he saw himself.

*Help patients see good they do not see. It is there*.

##### 3.1.2.5 Discuss religion—perilous, again, though this may be or at least seem to be contraindicated

Providers are commonly advised that they should not discuss religion with their patients, but, rather, have them discuss these topics with clergypersons or pastoral counselors since they are more equipped and thus prepared to address patients’ spiritual concerns. Nevertheless, for many patients, their providers not being willing to discuss these issues with them is a deep emotional gap and a loss. Providers discussing their religious beliefs with them can be, however, uniquely uplifting. These discussions can even undo the suicide-generating guilt that patients may feel due to their religious beliefs.

As an example, married and partnered patients may believe that merely thinking of having sex with another person may result in their eternal damnation, much like, perhaps, the parents who feared that if they gave their child unnatural pain meds, this would go against God’s will. The above patients are devoutly religious. They may believe that if they just find another person to be attractive, this may have a similar outcome.

Providers may generally ask in this context whether such patients may see any religious distinction between what feelings they spontaneously have and how they then act in response to these feelings. Providers may ask them then if they believe that their God would judge and condemn them for having feelings they cannot choose or control. Providers merely asking these questions may serve to lessen such patients’ self-condemnation. By just asking these questions, providers may also evoke new thoughts. These questions may then be enough to alter or significantly lessen these patients’ conviction that they, unequivocally, may be damned for having feelings that they cannot control.

Discussions of religion can help undo unbearable self-condemning thoughts. This can move patients to imagine that in some ways they have put together self-harming ideas that may be wrong (62–64). These discussions can also, like the father condemning himself because his daughter was getting a divorce, enable such patients, then, to see themselves in a new, more positive light, even when this is unprecedented. I think here of patients who have suffered harm to themselves in efforts to help others. I recall, for example, a patient who helped others during a lunch break, came back late, and as a result of this, lost her job.

There are much more profound examples. I think here of the philosopher Simone Weil. During World War II, she said that she wanted to go with others behind enemy lines as a way to help end the war, though if she and others did this, they would most likely all have been killed (65). Providers can in these cases inform these patients that in their quest to help others, they may be enacting the highest goals in many religions—their sacrificing themselves for others. If patients have Christian convictions, providers can even speak of what they do and have done as in their essence comparative to acts of Jesus or Saints. This may be, to these patients, uniquely healing.

*Discuss religion. Even tell patients when they seem saintly.*

##### 3.1.2.6 Support patients’ taking responsibility—even when this is only 1% up to them

All providers encourage their patients to take responsibility for what they do and have done—even if they can only do better in their present and future. Thus, patients who have committed even horrendous crimes may still take responsibility for what they do from this point on. They may, knowing this, still derive feelings of self-worth as they go forward with their lives. Providers should tell them this. These patients may object in a given instance, saying that their spouse or partner, for instance, has been and is at fault 99% of the time. They may be right. They still can take responsibility, however, for whatever was or is within their control.

I recall, for instance, a woman who fumed because, for the first time ever, her husband threw a foam cup at her. He might have a newly acquired impulse control problem, she mused, even early dementia. What was missing from her account was, however, what she might have done, no matter how small, to elicit this. She could not see any way in which she too may have contributed to his doing this. As it turned out, he had tried to help her, asking her if she wanted to “sit” and she had misheard this word. She scowled. “I’m *not* angry”, she angrily said.

Providers’ ultimate goal here and one that they should tell patients is that they should free themselves in these negative interactive instances from seeking to assess who is to blame and assigning relative percentages of blame for each party. Each person’s task is rather to identify how they may have contributed and then to change this.

To whatever degree each contributed, they should not seek to make excuses but rather apologize, see if they can make amends, and look to how they can avoid repeating this in the future. Their self-worth continues to exist though. They, like all of us, will be vulnerable to harming others, both intentionally and unintentionally. Providers should also tell them this.

A second goal in these instances is for each person feeling harmed to not only not judge the other but also to seek to understand the other party’s feelings, much as we discussed above when we considered how providers might optimally respond when patients fight with an edge in their voice. Patients then can strive to validate some aspect of the other party’s feeling upset just as providers can, as we discussed above. The wife above could acknowledge, for example, how it would have been particularly hard for her husband to have experienced her angry reaction when he was just trying to help.

*Stress patients facing up to what they do. No excuses*.

##### 3.1.2.7 Challenge patients’ insistence on justice. What people feel is usually what is much more important

This need for patients to place others’ feelings above what they see as justice may arise in many guises. Patients commonly adhere to moral principles they feel they must not compromise, notwithstanding the devastating effects to others and themselves from their doing this that hopefully they can foresee. Providers often must address this so that their patients can see this and then make better choices for both others and themselves.

The prototypic example of this harm is grandparents who cannot resist telling their adult children, now parents of their grandchildren, what, in these grandparents’ view, their children, as parents, are doing wrong. These grandparents may be right. Discipline causes children, for example, some stress, but this may benefit these children later on by equipping them with greater resilience. Too harsh discipline may, though, be harmful.

Regardless of the extent to which grandparents are or are not right, however, their intervening too much or too often may have a negative outcome for all parties. Their adult children may ever increasingly distance themselves from these grandparents such that they lose what is most precious to them—their being able to be with their children and grandchildren. “But, we are *right*!” these grandparents may protest. This may be the case, but the loss they risk is clear.

*Tell patients that being right and respecting others’ feelings may conflict.*

##### 3.1.2.8 Caring more for individual patients versus treating greater numbers of patients

We have left out of the above discussion providers’ feelings, though these clearly may radically both determine and alter patients’ outcomes. A raised eyebrow, again, may cause harm. Contrariwise, providers grimacing in their own pain in response to patients’ sharing their pain may convey the most genuine concern.

Providers may come to feel more for their patients over time as if they were family. Then, they may have an ethical conflict—whether to continue to add quality to these same patients’ lives by continuing to see them or to stop seeing them to see more, different patients, which in net effect could achieve greater utility or at least, so their institution, if they practice within one, may think. Their committing themselves more to one or a few patients may have more care and more respect for these patients’ dignity, as might caring more for family members than for others.

This deontological value of respecting dignity is not consequence-based. Thus, these two kinds of values cannot be weighed quantitatively against each other. It may, though, be that caring and dignity for one or a few patients should justifiably outweigh utilitarian concerns though often since resources are limited, providers treating more patients will be urged as warranting greater moral weight.

I recall seeing an older patient with severe autism. He had a guardian and had been homeless for much of his life. He related how terrible this had been for him throughout every session. Once he started, he would not stop. So pressured was his speech. I could hardly get a word in edgewise.

I imagined after a time that I should be seeing him less often and, perhaps, use my skills to try to help greater numbers of other patients. We do not have endless medical resources and providers to spare, I mused. Just then, as if he had read my mind—as conceivably he may have—he said, out of the blue, that his meetings with me were the only meaningful moments he had in his life and were all he looked forward to. I continued to see him as often as I had.

*Question utility. Consider giving priority to individual patients.*

### 3.2 More controversial interventions

Many of the above interventions are controversial, but there are many, of course, more controversial than these. Here, I shall note three. They illustrate, by extension, the need for providers to at least think of considering going outside their usual “boxes”—as prescribed by both their professions and empirical evidence. This may be necessary to meet particularly the needs of patients whose symptoms make them “outliers” at the margins of more general guidance spectrums. Their needs may require *opposite approaches* for a panoply of reasons.

#### 3.2.1 Look to the future—even with “ropes”

A first ground for considering more controversial actions may stem from looking at patients’ longer-term needs and futures as opposed to only their shorter-term interests. The two may conflict. An instance causing the most anguish for providers is when patients are considering suicide but are adamant that they do not want to be admitted to a hospital (66, 67). We, and we hope they, at some level, want above all to live on and stay alive (68, 69). Nevertheless, at this same time, if providers go against their wishes and involuntarily hospitalize them, this may jeopardize these patients living on throughout their futures (70, 71).

They and we cannot know whether or not they might have a sudden, overwhelming impulse to take their lives, even if they have never had such an impulse before. Still, we may do better for these patients in the long run by not hospitalizing them under these conditions, difficult though this may be for hopefully only a short time for these patients and us (72, 73).

A patient coming to see me had suicidal thoughts. She had already bought a rope with which she could hang herself. This alone may for many or most providers tie their hands, making their hospitalizing these patients no longer discretionary since they have gone beyond just thinking how they would end their lives to acting based on this plan. She, though, did not want to be hospitalized. Thus, we came to an alternative agreement. I would call her every 4 hours the remaining day and then through subsequent days until and unless she said she needed less frequent contact. Our backup plan between these hours in case she needed me but could not reach me was to call 988 or 911 or go to an emergency room. As it turned out, we could resume a usual meeting schedule within a week and did. She, I should add, as a follow-up some time later after she was no longer seeing me called to tell me of a weekend workshop she had attended that she thought I might want to recommend to others. Her calling to tell me this seemed to me a gift like the above patient’s telling me how to best hammer a nail. She appears to have flourished. She may, though, not have if I had involuntarily hospitalized her.

*Think long term, value patients’ wants, and perhaps, together, bear uncertainty.*

#### 3.2.2 Respect first, in spite of the possible price. The end result may paradigmatically then be optimal!

There is again an almost inviolable professional obligation to consult with families and sometimes also friends when a patient is suicidal to get greater, possibly life-saving, additional information. Providers contacting these loved ones then may or may not tell them before they ask them questions what may be the likely or possible consequences of the information that they provide—like involuntary hospitalization. In response to this “warning”, some may say nothing. Others who want to help save their loved ones’ lives may then just go ahead.

A similar issue arises for forensic providers wanting to ask loved ones what they know about alleged offenders to help them determine whether, at the time of a crime, these persons were or were not criminally insane. This same warning may stymie these outside parties from speaking.

Providers always, of course, already warn patients that they may not respect their confidentiality if they think they pose an undue danger to themselves or others. Paradoxically, patients may respond to these and other such warnings by feeling *greater* trust. This greater trust may come about because providers have respected them by sharing this, particularly because they shared this, knowing that this could curb these patients’ sharing of information.

*Consider so informing or warning, though this could harm the patient.*

#### 3.2.3 Risking being and being perceived as paternalistic

Providers should consider helping patients whenever and in whatever way that they can (28, 74, 75). To best illustrate this, I will give examples of patients both wanting to live and wanting to die.

An elderly patient wanted to live on at any cost though he had “untreatable” cancer. He had tried three experimental treatments unsuccessfully. He then heard of still a fourth, offered in another country, but to do this, he would have to leave his children and grandchildren whom he much loved and who much loved him. He would be alone then for 3 months. His provider wondered. Should he merely respect this or should he ask, “Are you sure? Would it be okay for us to discuss this more?”

A second patient due to diabetes had lost a leg and was about to undergo surgical amputation and lose her remaining leg if she was to be able to continue to live. She preferred to die. Her provider in this case debated whether to ask her a similar question to the one posed above: “Could we discuss this more? Could we see if there might be reasons we might discover that might move you to want to continue to live?”

Both questions are “conventionally” paternalistic. They each involve these patients’ providers questioning what these patients already had come to feel certain that they want, as opposed to their not questioning these patients and accepting their deciding questions wholly on their own. These providers risk these patients seeing them as disrespecting them by suggesting that these patients might want to discuss their decisions more with them. These providers asking these questions may, however, increase these patients’ autonomy over the longer run. Is this then impermissibly paternalistic? Or is this not the highest standard of care?

One provider reported that he felt “weird” going this extra mile. I recall seeing a patient like the one above. She too needed a second leg amputation due to diabetes. I respected her autonomous choice to refuse a second amputation. I, decades later, *now* feel weird in a different way. I regret not questioning her more at that time.

*Add to patients’ thinking, whether or not this is paternalistic.*

## 4 Conclusion

Most of the approaches presented above potentially may add to providers’ skills in treating patients with mental health needs worldwide. The core challenges to providers discussed above include providers listening for their own ambiguity and reducing the risks this ambiguity may bring about. They also include offering to support patients and families to pursue whatever it is *they* want, even when, personally, providers wholly disagree (76, 77). This latter approach opposes providers always giving priority to their own moral views, and thus, their seeing their doing this as in all cases ethically preferable. General approaches highlighted are providers always validating first and even when patients verbally fight, though this effort to heal the relationship under these circumstances violates what many providers might see as a mandatory practice—setting boundaries. Specific interventions include providers abandoning neutrality to not abandon their patients emotionally. This again departs from some providers’ adamant view. I suggest that providers may justifiably consider seeing patients longer even though they could instead help more patients. More extreme controversies considered here include not hospitalizing patients who are suicidal and even not calling their loved ones to get more information since either or both may in the long run *further* endanger these patients’ lives. Finally, we may question our patients both when they want to live and want to die.

There are here many common ground rules to accept or reject. These may test us. They may, too, change the destinies of patients like Tina. Globally.

## Author contributions

EH: Conceptualization, Formal analysis, Writing – original draft.

## Author disclaimer

The opinions and assertions expressed herein are those of the author(s) and do not reflect the official policy or position of the Uniformed Services University of the Health Sciences or the Department of Defense. Neither I nor my family members have a financial interest in any commercial product, service, or organization providing financial support for this research. References to non-Federal entities or products do not constitute or imply a Department of Defense or Uniformed Services University of the Health Sciences endorsement.
